# The co‐occurrence of antibiotic resistance genes between dogs and their owners in families

**DOI:** 10.1002/imt2.21

**Published:** 2022-05-04

**Authors:** Ruonan Zhao, Jie Hao, Jintao Yang, Cuihong Tong, Longfei Xie, Danyu Xiao, Zhenling Zeng, Wenguang Xiong

**Affiliations:** ^1^ Guangdong Provincial Key Laboratory of Veterinary Pharmaceutics Development and Safety Evaluation, College of Veterinary Medicine South China Agricultural University Guangzhou Guangdong China; ^2^ Guangdong Laboratory for Lingnan Modern Agriculture Guangzhou Guangdong China; ^3^ National Laboratory of Safety Evaluation (Environmental Assessment) of Veterinary Drugs, College of Veterinary Medicine South China Agricultural University Guangzhou Guangdong China; ^4^ National Risk Assessment Laboratory for Antimicrobial Resistance of Animal Original Bacteria, College of Veterinary Medicine South China Agricultural University Guangzhou Guangdong China

**Keywords:** companion animals, dogs, fecal resistome, gut microbiome, metagenomic

## Abstract

The intimate relationship between humans and companion animals causes a unique and critical aspect of antimicrobial resistance in humans. However, a comprehensive analysis of antimicrobial resistance between companion animals and their owners is lacking. Here, we chose 13 owned dogs and 16 owners as well as 22 kennel dogs to analyze the effect of an intimate relationship between owned dogs and owners on their gut microbiome, antibiotic resistance genes (ARGs), and mobile genetic elements (MGEs) and study the correlation of antimicrobial resistance between dogs and their owners in families by metagenomics. Dog gut microbiota had a higher abundance and diversity of ARGs while owners had a higher diversity of taxonomy. In the owned dog gut microbial community, ARG and MGE compositions were significantly more similar to the owner's gut microbiota than those of others. From the perspective of families, there was a strong correlation between macrolide resistance genes between dogs and their owners. In conclusion, our study demonstrated the correlation of ARGs between dogs and their owners at a community‐wide level. These findings can alarm the use of antibiotics in companion animals, which implies the potential to harbor antimicrobial resistance and threaten public health.

## Highlights

- The higher abundance of Gammaproteobacteria contributed to a higher resistance load in dogs.
- The intimate relationship affected the intestinal microbial community of dogs and their owners.
- The shared bacterial community caused the co‐occurrence of antibiotic resistance genes in families.

## Graphical abstract

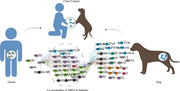

## INTRODUCTION

Antimicrobial resistance (AMR) is a “global public concern” regarded by numerous well‐known organizations, like the World Health Organization and Centers for Disease Control and Prevention [1]. AMR and the infection caused by antimicrobial‐resistant pathogens pose a serious global threat of growing concern to the human, animal, and environmental health [2]. The pathogens causing these infections can acquire antibiotic resistance genes (ARGs) in a process termed horizontal gene transfer (HGT). The HGT mediated by mobile genetic elements (MGEs) are important drivers of ARG dissemination, which facilitate the transmission of ARGs to recipient hosts and thus generate new antimicrobial‐resistance bacteria [3, 4]. MGEs, such as plasmids, integrative and conjugative elements (ICEs), transposons, and integrons, can move within or between DNA molecules and transfer between bacterial cells [5].

An increasing number and range of species of companion animals are kept in close interaction with human beings in industrialized societies. Figures vary considerably around the globe, but companion animals are considered by their owners to be an integral part of the family unit [6]. Owners can gain significant improvements in health and wellbeing from the ownership of companion animals or even interaction with them [7]. What's more, the past 10 years have witnessed the value companion animal cancer patients have within drug development and optimization efforts for human  [8]. However, companion animals have been described as potential reservoirs of AMR. It is known that owners often share the same environment with their companion animals that can be carriers of antibiotic resistance microorganisms (ARMs). Especially from a One‐Health perspective, companion animals might be a source of transmission of resistance genes and/or resistant bacteria to their owner [9, 10]. The major ARMs from companion animals that may directly or indirectly cause adverse health effects in humans are carbapenemase‐producing Enterobacteriaceae, extended‐spectrum beta‐lactamase (ESBL) Gram‐negative bacteria, methicillin‐resistant *Staphylococcus aureus* (MRSA), and vancomycin‐resistant *enterococci* [11]. MRSA can be passed between pet animals and owners with the possibility [12, 13]. The spread of ARMs, ESBL *Enterobacteria*, challenges human and veterinary healthcare settings worldwide and poses a public health threat [14]. The European Medicines Agency has already addressed the lack of knowledge about factors and transmission routes for the transfer of AMR between companion animals and their owners [15].

**Figure 1:**
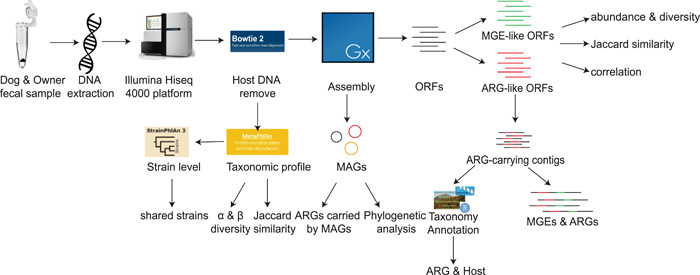
Analysis pipeline of this study. Metagenomic sequencing of fecal samples from 16 owners, 13 owned dogs, and 22 kennel dogs. Together with metagenomic sequence of 22 Hadza downloaded online, all metagenomic sequence was removed from host DNA for assembly and analysis of the microbial community. The ORFs were searched against resfinder and a custom MGE database to identify ARGs and MGEs. The ARGs and MGEs were used to analyze the sum abundance, diversities, Jaccard similarity, and correlation among different groups. Furthermore, we deciphered the bacterial host of ARGs and annotated the surrounding MGEs. ARG, antibiotic resistance gene; MAG, metagenome‐assembled genome; MGE, mobile genetic element; ORF, open reading frame

The gut harbors diverse AMR determinants, which have collectively been termed the “gut resistome” [16]. Currently, there are only a limited number of studies characterizing the gut microbiomes of companion animals [17]. Through metagenomic analysis, dog gut microbiomes were found to be similar to humans in gene content and response to diet compared to other mammals [18]. The previous studies of AMR between companion animals and their owners were mainly based on a culture‐dependent method [19]. However, the culture‐based studies to comprehensively characterize the ARMs in companion animals and human gut still remain to be improved. Belas found that the resistome of companion animals was similar to those of humans living in close contact by using a genomic approach [20]. However, it only focused on the ARGs associated with clinically important bacteria and was limited in the ability of polymerase chain reaction to detect ARGs as the sequences are not complementary to the primers and probes. Recently, several studies used metagenomic assembly and binning methods to reveal new insights into the linkage of AMR and microbial community [21, 22]. Here, we use metagenomic assembly and binning methods to study the effect of the close contact between owned dogs and owners on their gut resistome and decipher the prevalence and potential mobility of ARGs between them. The research pipeline was shown in Figure 1.

## RESULTS

### Dog gut had high levels of ARGs

**Figure 2:**
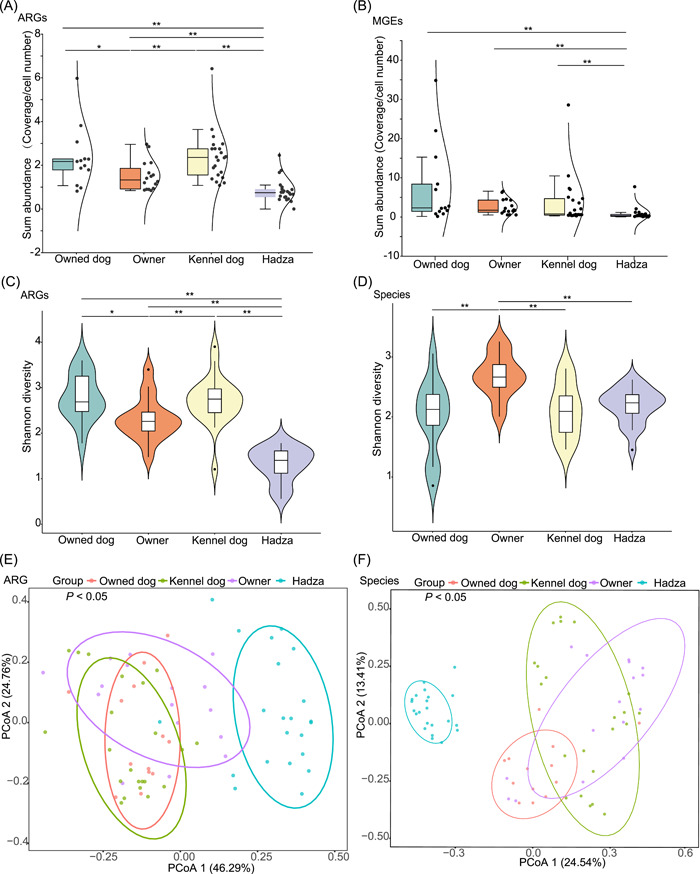
Sum relative abundance and diversity of ARGs and MGEs in owned dogs, kennel dogs, owners, and Hadza. (A) ARG sum relative abundances. (B) MGE sum relative abundances. (C) Shannon diversity of ARGs. (D) Shannon diversity of microbial taxa. (E) PCoA of gut ARGs. (F) PCoA of gut microbiomes. Owned dogs (*n* = 13), owners (*n* = 16), kennel dogs (*n* = 22), Hadza (*n* = 22). ARG, antibiotic resistance gene; MGE, mobile genetic element; PCoA, principal coordinates analysis. *p < 0.05, **p < 0.01

We compared the ARG, MGE, and taxonomy composition among four groups: owned dogs, kennel dogs, owners, and Hadza to discover their difference in these three aspects. Owned and kennel dogs' gut microbiomes had a higher abundance of ARGs than the gut microbiomes of owners and Hadza. However, the abundance of MGEs did not significantly differ among owned dogs, kennel dogs, and owners (Figure 2A,B). The same situation was also shown in the diversity of ARGs and MGEs. Nevertheless, the taxonomic diversity was significantly lower in dogs than in owners (Figures 2C,D and S1A). Hadza not only had the lowest abundance of ARGs and MGEs, but the shannon diversity of all aspects including ARGs, MGEs, and taxonomy was also the lowest.

Principal coordinates analysis was done to cluster samples using relative abundance on the gut microbiomes, ARGs and MGEs. We observed that samples of owned dogs and owners can be clustered into one group not only in their ARG but also in their MGE composition (*p* > 0.05, Adonis), while in their microbial community, they were separated (*p* < 0.05, Adonis) (Figures 2E,F and S1B). The situation was also shown in the samples of owned and kennel dogs. The samples of kennel dogs and owners had differences not only in their ARG composition but also in their microbial community composition (*p* < 0.05, Adonis). Hadza harbored a microbial community, ARG and MGE profile distinct from the kennel dogs, owned dogs, and owners (*p* < 0.05, Adonis).

### Owned dogs' resistome and microbiota resemble those of their owners

**Figure 3:**
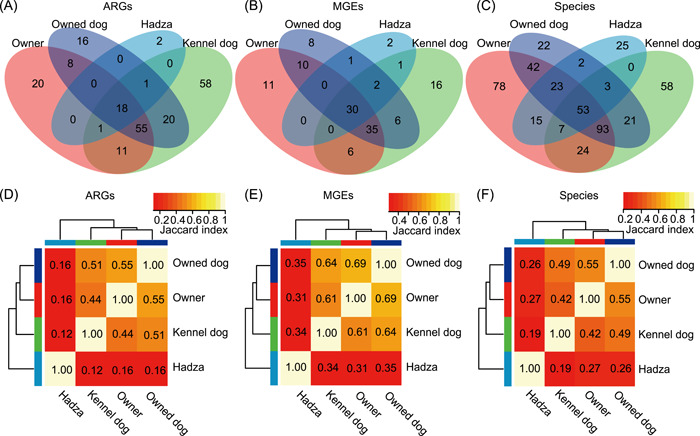
The relationship of gut ARGs, MGEs, and species among owned dogs, kennel dogs, owners, and Hadza. (A–C) The shared ARGs (A), MGEs (B), and species (C) among these four groups. (D–F) The distance matrix heatmap of gut ARGs (D), MGEs (E), and species (F). Species quantified by MetaPhlAn 2. ARG, antibiotic resistance gene; MGE, mobile genetic element

We counted their shared ARGs, MGEs, and species and further analyzed the similarity of the gut microbial community, and ARG and MGE compositions using the Jaccard index among the four groups. Owned dogs shared 70% of their ARGs, and 82% of their MGEs with their owners, and the ratio of shared ARGs and MGEs was 72% and 82% in owners, respectively. Kennel dogs had the highest diversity of ARGs and MGEs. However, kennel dogs only shared 57% of their ARGs with owned dogs and the shared ARGs between kennel dogs and owners only accounted for 52% of the total ARGs. The ratio that kennel dogs shared MGEs with owned dogs and owners was also lower than the shared MGEs between owned dogs and owners (Figure 3A,B). Furthermore, 81% of the species found in owned dog's gut were also detected in the owner's gut (Figure 3C). The Jaccard index of ARGs, MGEs, and gut microbial community between owned dogs and owners was 0.55, 0.69, and 0.55, respectively. There were higher similarities in the gut microbial community and ARG and MGE compositions between owned dogs and owners than in other pairs (Figure 3D–F).

### The comparison between dogs and their owners in families

**Figure 4:**
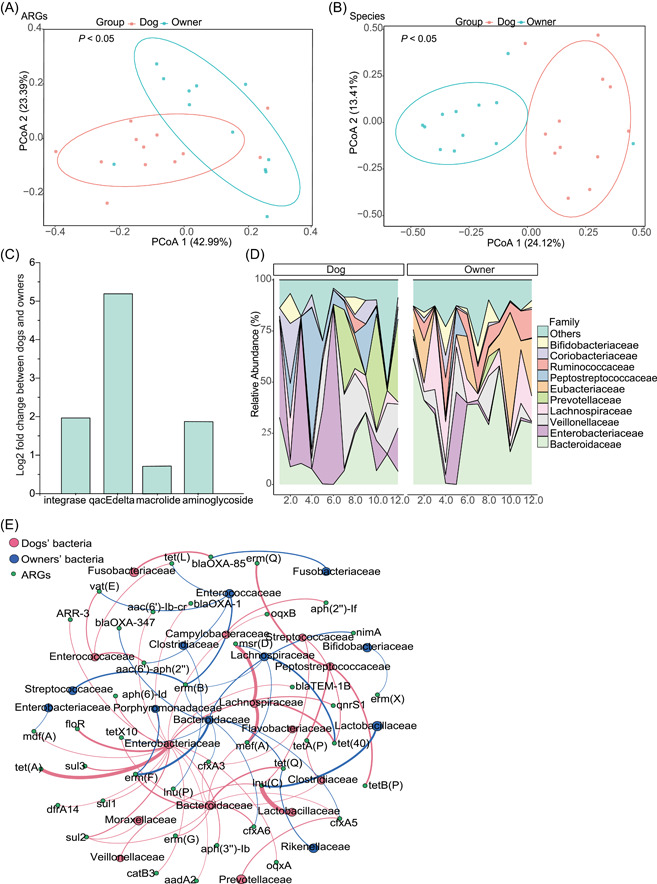
The comparison of ARG, MGE, and microbiome composition between dogs and their owners in families. (A) PCoA of gut ARGs. (B) PCoA of gut microbiomes. (C) Differences in the relative abundance of ARG and MGE types. (D) Top 10 families. The remaining families were represented as others. (E) ARGs and their bacterial hosts at the family level. Only ARGs with a relative abundance over 0.01 were shown. The nodes were colored according to the modularity class. The size of the curves represented the abundance of ARGs carried by their hosts. Dog (*n* = 12) and owner (*n* = 12). ARG, antibiotic resistance gene; MGE, mobile genetic element; PCoA, principal coordinates analysis

We then picked 12 dog‐owner pairs from the perspective of the family to compare their gut microbial community, and ARG and MGE compositions. We also found that dogs had higher total abundance and Shannon diversity of ARGs with lower taxonomic diversity than their owners. Meanwhile, the abundance and Shannon diversity of MGEs did not show a significant difference between the two groups. However, we observed the difference with the previous result in the aspect of beta diversity, dogs and their owners had differences not only in their microbial community but also in ARG composition (*p* < 0.05, Adonis; Figure 4A,B), while in their MGE composition, there was no significant difference between them (*p* > 0.05, Adonis; Figure S2A). Differences in the ARG and MGE compositions between dogs and owners were also seen in ARG and MGE types (Figure S2B,C). In terms of ARG types, the top four ARG types in both dogs and owners were aminoglycoside, beta‐lactam, macrolide, and tetracycline. Their abundance accounted for 83% and 95% of the total ARGs identified from dogs and owners, respectively. The most abundant resistance type in both dogs and owners was macrolide and its abundance was higher in dogs than in owners. Moreover, aminoglycoside‐ and trimethoprim‐resistant genes were also higher in dogs than in owners. When at the MGE level, transposases constituted the most common MGE types in all samples, and integron‐associated integrases and qacEdelta were significantly higher in dogs compared to the owners (Figure 4C). At the class level, Gammaproteobacteria and Epsilonproteobacteria were more common in dogs while owners had more *Clostridia* and Deltaprotebacteri (Figure S2D). At the family level, Bacteroidaceae was the most abundant in fecal samples and its abundance was higher in owners than in dogs. Dogs had a higher abundance of Enterobacteriaceae and Peptostreptococcaceae (Figure 4D). Furthermore, we characterized the difference in fecal microbiota between groups and discovered indicator genera (*p* < 0.05) by using linear discriminant analysis effect size (Figure S3).

### Gut resistome was linked to microbiome composition

We explored the link between gut ARG, MGE, and microbiome composition through the Procrustes analysis. The result of Procrustes analysis indicated that ARG, MGE, and microbiome composition correlated significantly with each other, suggesting the microbial community composition structured the gut ARG and MGE composition in the fecal microbiome of dogs and owners (Figure S4). We checked and summarized which microbial taxa (seen in less than five samples) correlated with ARG and MGE sum abundance in dogs and owners by analyzing their Pearson correlation (Table S2). On the other hand, strong correlations between taxa, such as *Escherichia coli*, *Escherichia*, Enterobacteriaceae, and Gammaproteobacteria, and the total sum abundance of ARGs and MGEs were observed in both dogs and owners.

Meanwhile, we deciphered the bacterial host of ARGs by the taxonomic annotation of ORFs carrying ARGs. At the family level, Enterobacteriaceae was the predominant bacterial host and harbored the most diverse ARGs, including the majority of ARG types in dogs while Bacteroidaceae was the predominant bacterial host in owners (Figure 4E). At the class level, Gammaproteobacteria harbored most of the diverse ARGs in dogs while the ARGs from owners were mostly carried by Bacteroidia (Figure S5).

### The co‐occurrence of ARGs and strains in families

**Figure 5:**
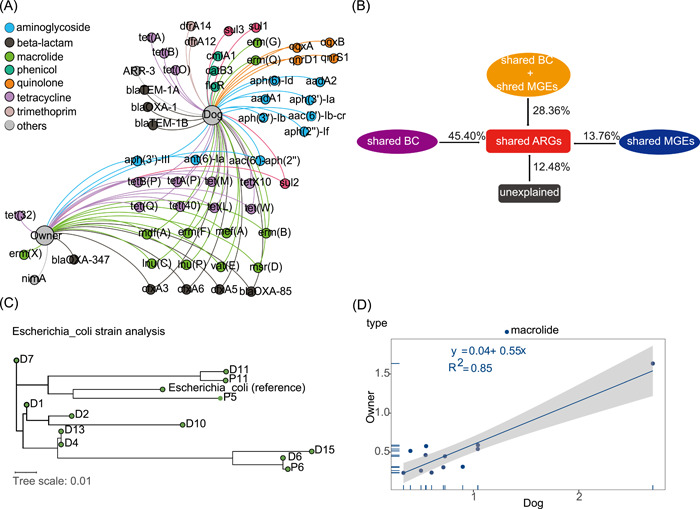
Analysis of shared ARGs and strains between dogs and their owners. (A) Bipartite network analysis depicting the shared ARGs between dogs and owners. Only the ARGs with a relative abundance over 0.01 were shown. The nodes were colored according to the modularity class and the size of curves indicated the number of samples harboring the ARGs. (B) Variation partitioning analysis differentiates the effects of the shared bacterial community and shared MGEs on the shared ARGs. (C) Phylogenetic tree of *Escherichia coli* at strain‐level using StrainPhlAn 3. The red box means dog and owner from the same family. The reference genome of *E. coli* is from *E. coli str. K‐12 substr. MG1655* (D) The correlation of ARG types between dogs and owners. ARG, antibiotic resistance gene; MGE, mobile genetic element

We analyzed the correlation of ARGs and shared strains between dogs and their owners and summarized the MGEs surrounding ARGs in the same contigs to explore the possibility of ARGs transmission in families. A total of 118 and 89 ARG subtypes were commonly detected in dogs and owners, respectively. More than half of the detected ARGs (61 ARGs) were shared by dogs and owners. These shared ARGs were widespread in the family environment, which had accounted for 88% and 96% of the detected ARGs of dog and owner samples in the aspect of the total abundance, respectively. We also found the abundance of shared ARGs was significantly positively correlated with their total sum abundance (*r* = 0.988 for owners and *r* = 0.925 for dogs, *p* < 0.01). A bipartite network analysis was adopted to present the shared ARGs between dogs and their owners and those unique ARGs in each group (Figure 5A). Most of the shared ARGs were resistance genes of commonly used antibiotics like beta‐lactam, aminoglycoside, and tetracycline, macrolide. Among the shared ARGs, the genes of *tet(Q)*, *tet(A)*, *lnu(C)*, *mef(A)*, *erm(F)*, and *erm(B)* (resistant to tetracycline and macrolide) took up the largest proportion in both dogs and owners. Relationships between shared bacterial community (at the class level), shared MGEs, and shared ARGs were studied using redundancy analysis (Figure S5B). To determine the contributions of the shared bacterial community and shared MGEs on the shared ARGs, variation partitioning analysis (VPA) was done and we found our selected factors explained 87.52% of variations in shared ARG profile. The interaction between shared bacterial community and shared MGEs contributed 28.36% to the shared ARG profile and the single shared bacterial community explained 45.40% of the variance, higher than that of shared MGEs (13.76%) (Figure 5B). We also utilized StrainPhlAn3 to study whether dogs and their owners shared strains and found 12 consensus strains of *E. coli* among all of the 24 fecal samples among which there were two pairs from the same families (Figure 5C). In addition, four consensus strains of *Klebsiella pneumoniae* could be constructed (Figure S6A). Furthermore, we analyzed the Pearson correlation of ARGs between dogs and owners. Although the total abundance of overall ARGs did not show correlation (*p* > 0.05), we found the high correlation of macrolide resistance genes (*r* = 0.92, *p* < 0.01) between dogs and their owners. The correlation was also seen in the ARG subtypes, such as *mdf(A)* and *tet(L)* (Figures 5D and S6B).

The ARGs were also significantly correlated with MGEs (Figure S4). The presence of MGEs surrounding ARGs shared among dog‐owner pairs was also investigated. All together 104 contigs containing at least an ARG and an MGE were assembled from the sample (Table S3). Of those 104 contigs, 6 contigs with the same arrangement of genes in the contigs were shared between a related pet‐owner pair and 35 contigs were shared between dogs and owners, the most common contig was the *tnpA* with a *cfxA3* resistance gene. It also revealed that several of the ARG‐containing contigs had their best taxonomic hit on *E. coli*.

### The metagenome‐assembled genomes reveal the representative bacterial communities and their ARGs

**Figure 6:**
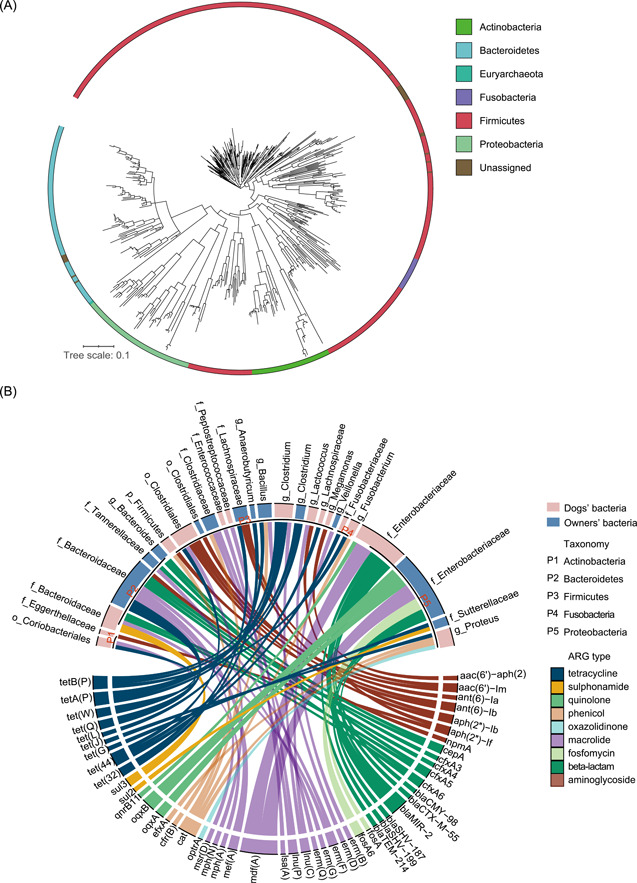
The analysis of MAGs. (A) Phylogenetic assignment of MAGs. Organisms are colored based on phyla. (B) The mapping relationship between the 58 ARG‐carrying MAGs and the carried ARGs. The length of the bars on the inner ring represented the total number of ARGs and MAGs, respectively. ARG, antibiotic resistance gene; MAG, metagenome‐assembled genome

The sequence composition‐independent genome binning was also conducted to analyze the representative bacteria and their carried ARGs. From 24 samples of 12 families, bacterial genomes were constructed with the combined assembly of filtered reads. After filtering low‐quality metagenome‐assembled genome (MAG), a total of 326 MAGs in owners and 215 MAGs in dogs were obtained. We combined all the MAGs from owners and dogs together to conduct the phylogenetic analyses. The phylogenetic tree revealed that they belonged to six bacterial phyla, including Actinobacteria, Bacteroidetes, Euryarchaeota, Firmicutes, Fusobacteria, and Proteobacteria (Figure 6A). Seventy MAGs were classified at the species level, 58 were classified at the genus level, 224 were classified at the family level, 124 were classified at the order level, 6 were classified at the class level, 29 were classified at the phylum level, and the remaining 20 were only identified as bacteria because of the limitation of available related reference genomes.

Among the 541 recovered MAGs, a total of 58 MAGs (30 in dogs and 28 in owners) were identified as carrying ARGs (*n* = 108). The bacterial hosts of ARGs mostly belonged to the family Enterobacteriaceae in both dogs and owners (Figure 6B). The 58 MAGs carried nine ARG types and the four most frequently detected ARG types were macrolide, tetracycline, beta‐lactam, and aminoglycoside. Moreover, 21 MAGs (11 in dogs and 10 in owners) carried at least two ARGs. Two MAGs annotated as family Enterobacteriaceae in owners harbored five ARGs conferring resistance to beta‐lactam (*blaSHV‐187, blaSHV‐199*), fosfomycin (*fosA*, *fosA6*), macrolide (*mdf*(*A*)), and quinolone (*oqxA, oqxB*). In addition, a total of 13 MAGs (four in dogs and nine in owners) assigned to the family Bacteroidaceae were resistant to the most of ARG types, including aminoglycoside, beta‐lactam, macrolide, sulfonamide, and tetracycline.

We further predicted the ICEs and integrative and mobilizable elements (IMEs) in the ARG‐carrying MAGs to further estimate the transferability of ARGs (Table S4). A total of 13 T4SS‐type ICEs and 30 IMEs were identified in 22 ARG‐carrying MAGs, of which six MAGs contained both ICE and IME regions. For other components, seven IMEs in five MAGs were found to contain an oriT region, a short DNA sequence that is critical for conjugation. Moreover, the attachment sites (*attL* and *attR*) flanking most ICE and IME regions were observed, indicating their potential roles in site‐specific recombination events.

## DISCUSSION

The intimate contact between companion animals and their owners is a risk factor for AMR transfer. Especially, the ARMs reside on the dog's skin and mucosal skin might easily be transferred to owners. Furthermore, some resistance levels are worrisome, as a possible transmission of *mcr‐1*, resistant to colistin, from companion animal to human was reported [23].

In this study, we reported the comparation of the gut ARGs, MGEs, and microbiota among owned dogs and their owners as well as kennel dogs and Hadza. Current studies mainly used traditional bacterial culture methods to explore the AMR of dogs and humans. However, the limitations of the methods impose restrictions at the community‐wide level [24]. Using a metagenomic assembly and binning approach, we provided a comprehensive insight into the association of AMR between owned dogs and owners. Dog's gut had higher levels of ARGs while owners had higher levels of microbial diversity and they did not differ significantly in MGEs. The higher abundance of ARGs in owned and kennel dogs may be explained by the use of antibiotics. Antimicrobials are useful tools for the therapy of infectious bacterial diseases in pets; however, there were no specific guidelines for prudent antibiotics use in pets [25]. Antimicrobial use in small animals has been identified as one of the risk factors for colonization or infection with ARMs [26]. Furthermore, antibiotic‐resistant bacteria and ARGs can be from their food [27]. For kennel dogs, another possible cause is group housing.

Recent studies suggested that the shift from a natural and undomesticated lifestyle to cohabitation with humans has modulated the intestinal microbial community of domesticated dogs [28, 29]. There were many similarities between human and dog gut microbiomes compared to other mammals [18]. We found that owned dogs not only in aspects of gut microbiome but also ARG and MGE were more similar to the owners. Previous studies highlighted the important role of the environment in the gut microbiome as they found related humans had a more similar microbiome composition than unrelated humans [30, 31]. Likewise, gut microbiomes of genetically related dogs seem to be more similar to each other than those of unrelated dogs [32]. Furthermore, dogs living in households had different gut microbiome composition than dogs living in shelters, which demonstrated the living environment likely has an important influence on its composition [17]. Meanwhile, the close relationship between pets and owners influenced the owner‐associated microbial community. The presence of a dog within a family generally leads to higher microbial biodiversity of the owner's skin when compared to humans without pets [33]. Our results strengthened the notion that cohabitation and the close relationship between pets and their owners play an important role in gut microbiota modulation with repercussions on the health of both parties [34].

Considering the close relationship and the increased frequency of antibiotic‐resistant bacteria detected in humans and companion animals, new opportunities are created for interspecies transfer of AMR [35]. We studied the correlation of AMR between dogs and their owners in families. From the perspective of families, dogs and their owners also had similarities to ARG and MGE profiles. Macrolide, the highest ARG type in dogs and owners, is one of the top three most prescribed antibiotic classes in human medicine [36]. A total of 18 tetracycline resistance genes including *tetX*, the most widespread and dominant ARGs and typical for gut microbial, were detected with high abundance in dogs and owners. Their prevalence in dogs may be due to the situation that companion animal practitioners prefer human antibiotics for their better quality and easy availability [37]. There was a high use of broad‐spectrum antimicrobials and critically important antimicrobials for human medicine in companion animals [9, 38].

Gut microbiomes are a wide source of ARGs and potential reservoirs for pathogenic bacteria becoming more resistant [35]. ARGs can be transferred between different environments via specific bacteria, we used Procrustes analysis to explore potential HGT frequency among bacterial populations and found that ARGs, MGEs, and microbial communities correlated significantly with each other. Previous studies mainly used network analysis to explore the potential hosts of ARGs [39]. Nevertheless, these results based on mathematical statistics only revealed limited ARGs hosts and need to be further validated. Recently, several studies investigated ORFs of ARGs in assembled contigs [40]. Here, we figured out which microbial taxa significantly correlated with ARG and MGE sum abundance through correlation analysis and explored the potential hosts of the specific ARGs by annotation of ARG‐carrying contig. We found Gammaproteobacteria and Enterobacteriaceae contributed to a high resistance load and most likely harbored the majority of the most abundant ARGs. This could be identified by the theory that Gammaproteobacteria and Enterobacteriaceae are key drivers of the dissemination of important resistance mechanisms [11, 41]. Dogs had more Gammaproteobacteria including Enterobacteriaceae than the owners, suggesting there might be elevated rates of gene transfer in the dog gut with a higher abundance of those as they could promote the spread of ARGs. Considering that, the role of dogs in the family as reservoirs of resistant bacteria is concerning.

The shared ARGs accounted for the majority of the total abundance of ARGs and the categories referring to shared ARGs are of concern due to their prevalence. Our results suggested that shared bacterial community played an important role in the occurrence of ARGs in families. Utilizing StrainPhlAn3, we found that *E. coli* was the shared strain between dogs and their owners. In the terms of ARGs, we found that macrolide‐resistant genes and some specific ARG subtypes showed a strong correlation between dogs and owners. Several antimicrobial classes are used in humans and companion animals are the same, leading to an overlap of the detected ARGs [42]. The investigation of contigs containing ARG and MGE found that the same arrangements were shared by dogs and owners, suggesting these ARGs are usually associated with MGEs, which are important for AMR transfer between dogs and owners. Our results also suggested that there was a possibility of ARGs and MGEs dissemination between dogs and their owners in families. The ARG‐containing contigs had their best taxonomic hit to *E. coli*, indicating that many ARGs carried by the strain in the dog and owner gut are mobile. It was consistent with the result that *E. coli* had a strong correlation with the total sum of ARG and MGE abundance. Nevertheless, owing to the technical challenge of short read assembly of low abundance genes, the analysis based on gene annotations cannot distinguish between nucleotide level variants of the genetic elements [43–45].

Metagenomic binning will enable a better understanding of AMR and provide important insights into the mobilization of ARGs [22]. We also explore the host and the mobility of ARGs by the recovered MAGs. The family Enterobacteriaceae carried most of the ARGs, which was consistent with the above identification of the bacterial host of ARGs. As for the mobility of ARGs, it has been widely reported that ICEs play an important role as vehicles for the transfer of AMR between different bacterial species and genera [46, 47]. In addition to the ICEs, the chromosome‐borne IMEs were also found in some ARG‐carrying MAGs. In a review, IMEs were considered a likely reservoir of ARGs [48]. The MAGs carried multiple types of ARGs and ICEs should be of great concern because they can increase the mobility and risk of ARGs. Further studies are required to determine the causality and directionality of AMR between owners and them. To prevent the spread of AMR within households and establish an effective measure to control resistance need joint efforts from all sectors of the society.

## CONCLUSION

We first used the metagenomic assembly and binning approach to explore the prevalence and relationship of ARGs and microbiome between dogs and their owners. The higher abundance of Gammaproteobacteria and Enterobacteriaceae in dogs may contribute to a higher resistance load in dogs. The intimate relationship between owned dogs and their owners played a key role in their gut resistome and microbiome modulation with repercussions on the health of both parties. From the perspective of families, the shared bacterial community may be the main cause of the co‐occurrence of ARGs in families.

## METHODS

### Sample collection

Thirteen and 16 fecal samples of owned dogs and owners were collected from 15 families, the owner from family 10 had been sampled two times. To verify the impact of the intimate contact between owned dogs and owners on their gut resistome and microbiome, we collected the 22 fecal samples of kennel dogs, seldom in contact with humans, from three distinct kennels and 22 metagenomic sequence data of Hadza, a population with little antibiotic exposure and hardly any contact with dogs [49]. The detailed information of these samples was summarized in Table S1. All the collected fecal samples were kept at 4°C and transported to the lab within 2 days then stored at −80°C before DNA extraction.

### DNA extraction

For DNA extraction, 0.25 g of each sample was taken using the MoBio PowerSoil DNA isolation kit (MoBio Laboratories) following the protocol from the manufacturer. The purity and concentration of genomic DNA were determined by UV spectroscopy using a NanoDrop ND‐2000 instrument (NanoDrop Technologies). The DNA samples with OD260/OD280 under 1.8–2.0 and a concentration >50 ng/μl were used for metagenomic sequencing.

### Metagenomic sequencing and quality control

A library consisting of 300‐bp DNA fragments was constructed before sequencing. Each fecal sample was sequenced on Illumina HiSeq. 4000 platform using a strategy of index 150 PE (paired‐end sequencing). Raw reads with average quality scores <20 (Q20) or length <20 bp (L20) were removed using Sickle [50].

### Host DNA removal, metagenome assembly, and binning

A dog reference genome database was created using bowtie2‐build with the Canis lupus familiar is reference genome CanFam3.1 (NCBI accession number GCA_000002285.3) and a human reference genome database was created using bowtie2‐build with the Homo sapiens reference genome GRCh38.p13 (NCBI accession number NC_000001.11). The postfiltered reads were processed using the reference genome database to remove contaminant host DNA [51]. The raw reads and clean reads of each metagenome sample are summarized in Table S1. The clean reads were de novo assembled using CLC Genomics Workbench (version 10.01) with the default k‐mer size. Briefly, the MAGs were constructed using contigs by the binning algorithm “MetaBAT2” in the metaWRAP (v1.2.1) software [52, 53]. CheckM v1.1.2 with options lineage_wf, ‐x fa was used to assess the completeness and contamination of all MAGs [54]. Only the MAGs with an estimated quality ≥50% and contamination <10% were retained [55].

### Detection of ARGs and MGEs

The open reading frames (ORFs) were predicted using Prodigal version 2.6.3 [56]. The nucleotide sequences of the ORFs were searched against Resfinder Database [57] and a custom MGE database [44] for the identification of the ARG‐like ORFs and MGE‐like ORFs using BLASTN under an *E* value ≤ 10^10^. An ORF sequence with the best BLASTN alignment to ARG sequences cutoff ≥80% similarity and ≥70% query coverage was regarded as an ARG‐like ORF and an ORF sequence with the best BLASTN alignment to ARG sequences cutoff ≥90% similarity and the alignment at least 25 amino acids was regarded as an MGE‐like ORF [58]. The coverage of ARG‐like ORFs was calculated by mapping clean reads to the contigs with a minimum length coverage of 95% at 95% similarity and contigs minimum length ≥500 bp using the CLC Genomics Workbench [40]. The number of cells in each metagenome sample was calculated by stage on one of ARGs‐OAP v2.0 [59]. The coverage was normalized by the number of cells in each sample (copies/cell) and was defined as follows:

$$\text{Coverage}=\sum_{1}^{n}\frac{N\times 150/L}{C}.$$

Here, *N* belongs to the number of the reads mapped to ARG‐like ORFs, *L* is the target ARG‐like sequence length, *n* is the number of ARG‐like ORFs, 150 is the length of Illumina sequencing reads [40, 60], and  *C* means the cell number per sample.

The identification of ARGs carried by MAGs was also followed the above way. To further estimate the transferability of ARGs, the ICEs, IMEs in the ARG‐carrying MAGs were predicted by ICEfinder online tool with the default parameters [61]. Those elements carrying integrase, relaxase, and T4SS gene clusters were considered T4SS‐type ICEs, while those without T4SS but with integrase and relaxase were identified as IMEs [61].

### Analysis of gut bacterial community

Community profiling based on marker genes was done using MetaPhlAn 2 [62]. Strain‐level profiling and strain tracking analysis was done using StrainPhlAn3 [63]. We utilized StrainPhlAn3 to obtain consensus strains for the major ARMs originating from companion animals [11]. We also deciphered the bacterial hosts of ARGs by searching the protein sequences of the ORFs within the ARG‐carrying contigs against the NCBI NR database using BLASTP at an *E* value ≤ 10^−5^. The BLASTP output results were annotated using MEGAN (version 5, MEtaGenome ANalyzer) with the default parameters [64]. The taxonomic assignment of an ARG was determined by a majority vote if more than 50% of ORFs of the contigs were assigned to the same taxonomic rank using a customized R program for voting, otherwise, it was regarded as unclassified [65].

### Phylogenetic analysis and taxonomic analysis of MAGs

The retained MAGs were annotated to the genus using the Genome Taxonomy Database Toolkit (GTDB‐Tk) [66]. The phylogenomic analysis was performed using PhyloPhlAn 3.0 software based on a set of 400 conserved bacterial protein sequences [67].

### Statistical analysis

Statistical comparisons were done using nonparametric Kruskal–Wallis tests among four groups and nonparametric Mann–Whitney *U* tests between dogs and their owners in families. The multiple test correction was done through Bonferroni correction. A *p* value of less than 0.05 was regarded as statistically significant. Shannon diversity of taxonomic profiles, ARGs, and MGEs were calculated in the R (v4.0.2). The boxplot was performed by Origin (v9.6.5). Network visualization was conducted on the interactive platform of Cytoscape (v3.7.1) and Gephi (v0.9.2). The line regression was done on the website: https://hiplot.com.cn. The phylogenetic tree was plotted with iTOL v5 online [68]. The others were all performed in the R or ImageGP webserver [69].

## AUTHOR CONTRIBUTIONS

Ruonan Zhao analyzed the data and wrote the manuscript; Jie Hao collected the sample and did the laboratory work; Jintao Yang helped analyze the data and network visualization; Cuihong Tong, Danyu Xiao, and Longfei Xie joined the sample collection and laboratory work; Zhenling Zeng contributed to drafting the manuscript; Wenguang Xiong designed this study. All co‐authors contributed to drafting the manuscript and approved the final draft.

## CONFLICTS OF INTEREST

The authors declare no conflicts of interest.

## Supporting information

Supporting information.

Supporting information.

## Data Availability

The data and script used in this study can be found at https://github.com/zrn327/close-contact-affect-gut-bacterial-community. The datasets supporting the conclusions of this article are available in the NCBI Sequence Read Archive repository (accession no. PRJNA721002 for dogs and dog‐owners). Supporting Information Materials (figures, tables, scripts, graphical abstract, slides, videos, Chinese translated version, and update materials) may be found in the online DOI or iMeta Science http://www.imeta.science/.
