# Supplementary material for: The co‐occurrence of antibiotic resistance genes between dogs and their owners in families
Source: Imeta. 2022 May 4;1(2):e21. doi: 10.1002/imt2.21 (PMC10989978; doi:10.1002/imt2.21)
Supplement: Supplementary file 1 — Supporting information. [file IMT2-1-e21-s002.docx]

Supplementary Material for

**The co-occurrence of antibiotic resistance genes between dogs and their owners in families**

Ruonan Zhao, Jie Hao Jintao Yang, Cuihong Tong, Longfei Xie Danyu Xiao, Zhenling Zeng*, Wenguang Xiong*

Correspondence author: Zhenling Zeng (zlzeng@scau.edu.cn), Wenguang Xiong [(xiongwg@scau.edu.cn)](mailto:(xiongwg@scau.edu.cn))

**Figure S1.** The α and β diversity of MGEs. (A) MGE diversity with Shannon index. (B) PCoA of MGEs. ***P* < 0.01. MGE, mobile genetic element.

**Figure S2.** Abundant ARGs, MGEs and bacteria in dogs’ and owners’ gut. (A) PCoA of MGEs. (B) Most abundant ARG types. (C) Most abundant MGE types. (D) Top 10 classes. The remaining classes were represented as others. ARG, antibiotic resistance gene.

**Figure S3.** Genera differentially abundant in dogs and their owners. The right side showed the bacterial biomarkers on family level associated to dogs and owners.

**Figure S4.** Correlation analysis of ARGs, MGEs and microbial community based on Procrustes analysis. (A) Procrustes analysis depicted correlation between ARGs and microbial community. (B) Procrustes analysis depicted correlation between MGEs and microbial community. (C) Procrustes analysis depicted correlation between ARGs and MGEs.

**Figure S5.** ARGs and bactieral community. (A) ARGs and their host on class level. Only ARGs with the relative abundance over 1 % was shown. Triangles mean hosts and ellipses mean ARGs. The triangles were colored by modularity class. Solids represented ARGs with their hosts in dogs and dashes represented in owners. (B) Redundancy analysis (RDA) identified the correlation of shared bacterial classes (the relative abundance > 1% on average), shared MGEs (the relative abundance > 1% on average) and shared ARGs. (C1: Alphaproteobacteria; C2: Bacilli; C3: Bacteroidia; C4: Betaproteobacteria; C5: Clostridia; C6: Deltaproteobacteria; C7: Epsilonproteobacteria; C8: Erysipelotrichia; C9: Fusobacteriia; C10: Gammaproteobacteria; C11: Viruses).

**Figure S6. Analysis of strain and ARG subtypes**. (A) Phylogenetic tree of *Klebsiella pneumoniae* at strain-level using StrainPhlAn. The reference genome of *Klebsiella pneumoniae* is from *Klebsiella pneumoniae* *subsp. pneumoniae HS11286*. (B) The correlation of ARG subtypes between dogs and owners.
